# Liver Regeneration Following Thermal Ablation Using Nanocarrier Mediated Targeted Mesenchymal Stem Cell Therapy

**DOI:** 10.1007/s00270-024-03862-2

**Published:** 2024-11-06

**Authors:** Prasoon P. Mohan, Sapna Deo, Zhao-Jun Liu, Emre Dikici, Hugo Kaneku, Doyoung Chang, Monica Garcia-Buitrago, Hamed Jalaeian, Elnaz Zeynaloo, Yulexi Y. Ortiz, Yan Li, Shivank Bhatia, Omaida Velazquez, Sylvia Daunert

**Affiliations:** 1 Department of Interventional Radiology, University of Miami Miller School of Medicine, Miami, FL USA; 2 Department of Biochemistry, University of Miami Miller School of Medicine, Miami, FL USA; 3 Department of Surgery, University of Miami Miller School of Medicine, Miami, FL USA; 4 Department of Interventional Radiology, UMHC-SCC, 1475 NW 12th Ave., Miami, FL 33136 USA

**Keywords:** Thermal ablation, Liver regeneration, Stem cells, Nanocarrier

## Abstract

**Purpose:**

To test the efficacy of nanocarrier (NC) mediated mesenchymal stem cell (MSC) therapy for liver regeneration following thermal ablation of porcine livers.

**Materials and Methods:**

Liver radiofrequency ablation was performed in 18 swines divided into MSC, MSC + NC and control groups. The test groups received infusion of MSC or MSC + NC labeled with enhanced green fluorescent protein (eGFP) via hepatic artery. MSC + NC group had MSCs coated with dendrimer nanocarrier complexed with I-Domain of lymphocyte function-associated antigen-1 (LFA-1). Nanocarriers direct homing of MSCs by binding to its counterpart protein, intercellular adhesion molecule-1 (ICAM-1), which is overexpressed at the periablation margins from inflammation. Ablation cavity reduction by CT volumetry was used as surrogate marker for liver regeneration. Cell proliferation was assessed with Ki67 and HepPar-1 stains. GFP identified MSC derived cells.

**Results:**

Total number of ablations in control animals were 13 across 4 animals. In the MSC group, there were 23 ablations across 6 animals, and in MSC + NC group there were 21 ablations across 6 animals. Ablation cavity volume reduction from day 0 to 30 were 64.4 ± 15.0%, 61.5 ± 12.9% and 80.3 ± 9.4% for control, MSC and MSC + NC groups, respectively (MSC + NC vs MSC: *p* < 0.001, MSC + NC vs. control: *p* = 0.001). GFP^+^ cell count at margins was 426.8 ± 193.2 for MSC group and 498.6 ± 235.2 for MSC + NC group (*p* = 0.01). The mean Ki67 and HepPar-1 staining at margins were 9.81 ± 4.5% and 6.12 ± 4.2% for MSC + NC group versus 7.59 ± 3.7% and 5.09 ± 3.7% for MSC group, respectively (*P* < 0.001 and *P* = 0.09, respectively).

**Conclusion:**

Nanocarrier-mediated MSC therapy promotes liver regeneration by engrafting MSCs at ablation margins, potentially making liver-directed therapy viable for patients with severe liver dysfunction. This technology may also benefit other solid organs.

**Graphical Abstract:**

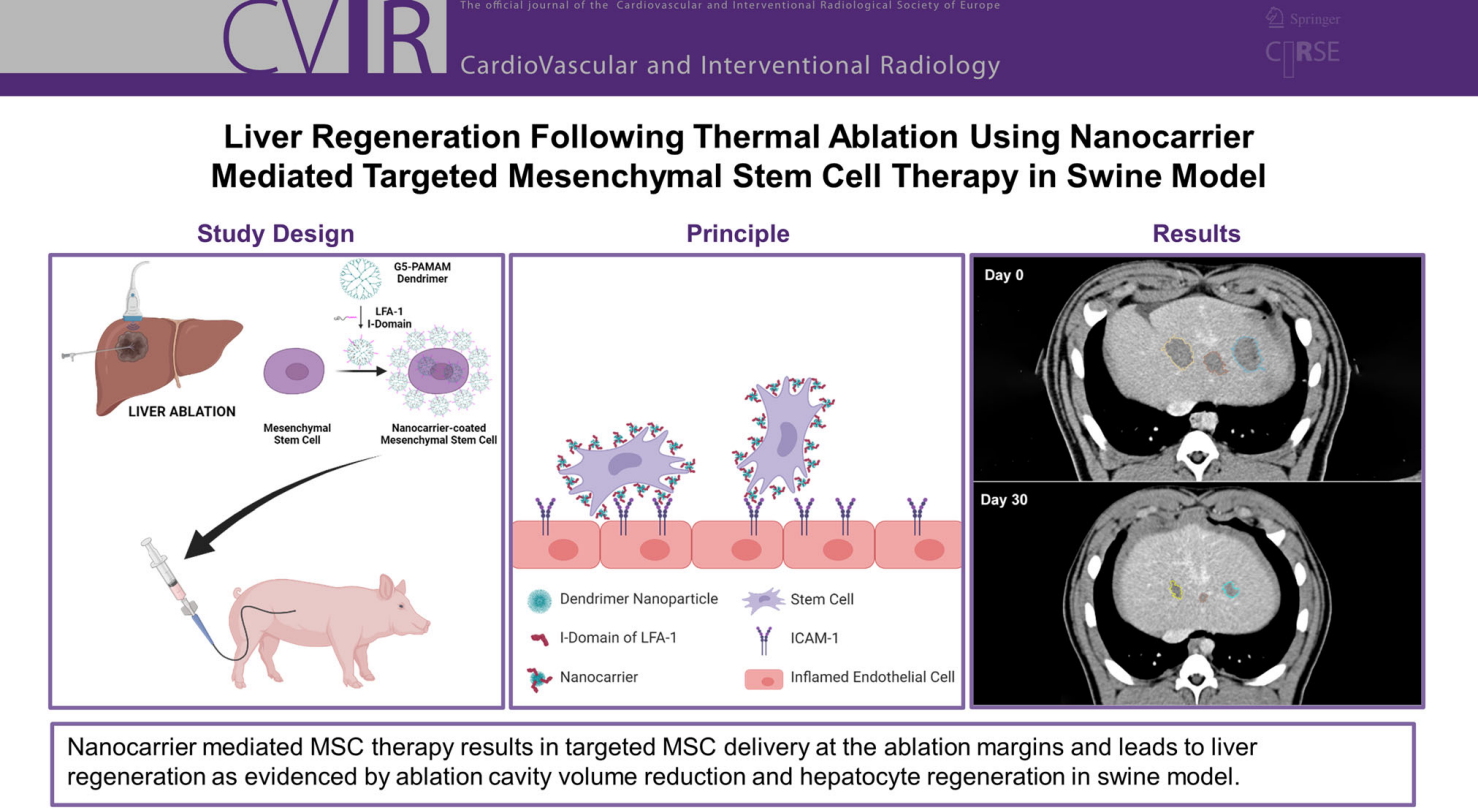

**Supplementary Information:**

The online version contains supplementary material available at 10.1007/s00270-024-03862-2.

## Introduction

Cirrhosis with associated liver dysfunction is present in 80–90% of hepatocellular cancer (HCC) [1]. Tissue regeneration using stem cells can potentially restore liver functions following liver-directed therapies. Bone marrow-derived mesenchymal stem cells (MSC) are used to promote tissue regeneration in organs, including the liver [2–4]. However, targeted delivery and homing of MSCs to the tissue that needs repair remains a challenge. Systemic venous delivery and intra-arterial delivery may result in distribution and engraftment of stem cells in random organs [5].

**Fig. 1:**
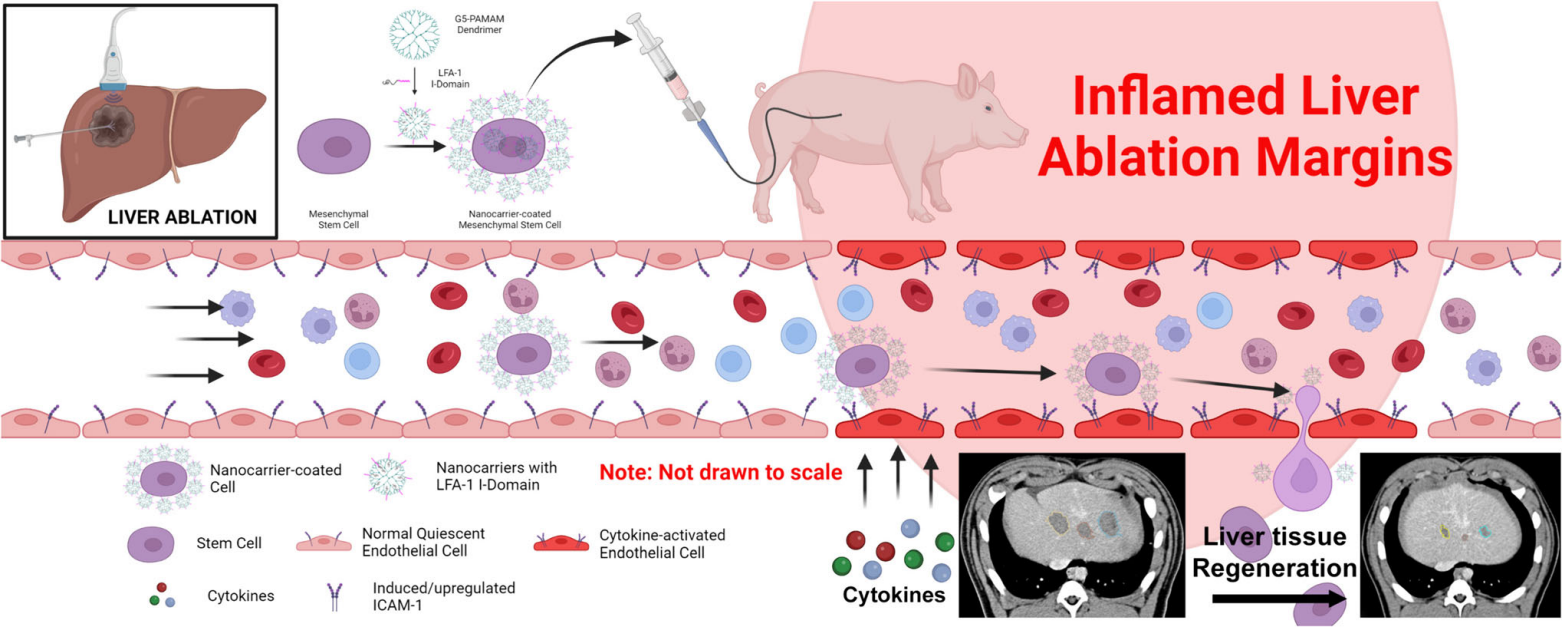
Graphical representation of the experiment. Following liver ablation, nanocarrier coated stem cells are infused into the liver via the hepatic artery. Nanocarriers are composed of dendrimer nanoparticles which are complexed with specific adhesion molecules (LFA-1). These are then coated on the surface of the mesenchymal stem cells. The adhesion molecules on the nanocarriers bind with the specific inflammatory marker (ICAM-1) on the activated endothelial cells at the inflamed ablation margin and docks the stem cells, which will in turn extravasate into the tissues. The stem cells promote liver regeneration at the ablation margins and decrease the ablation cavity volume

The aim of this pilot study is to test the safety and efficacy of targeted delivery and homing of MSCs using this nanocarrier-mediated system for liver regeneration following thermal ablation. The nanocarriers are made of dendrimer nanoparticles which are complexed with specific adhesion molecules (proteins). These nanocarriers on the cell surface help to direct the infused MSCs to their target destination. The specific adhesion molecule on the nanocarrier can recognize and bind with its counterpart adhesion molecule expressed on the activated endothelium in the injured tissues (Fig. 1). Once anchored on the activated endothelium, MSCs extravasate and home in the targeted tissues and promote regeneration (Fig. 1). The effectiveness of this delivery technique using E-selectin/ligand pair-mediated cell–cell interaction for directing MSC homing in mice wound healing has been previously demonstrated [6]. The targeting system tested in the current study utilizes the interaction between intercellular adhesion molecule-1 (ICAM-1) which binds to its counterpart adhesion molecule, the I-Domain in lymphocyte function-associated antigen-1 (LFA-1) [7, 8]. Radio frequency ablation (RFA) induces a time-dependent inflammatory response at the perimeter of the ablation zone, which peaks at 24 h [9]. This inflammatory rim around the ablation zone is the target for MSC delivery, where ICAM-1 is upregulated in endothelial cells in response to thermal injury [10, 11]. The I-Domain of LFA-1 on the nanocarriers attached to MSCs can bind with its counterpart adhesion molecule-ICAM-1 on the endothelium and direct its homing[12, 13].

## Materials and Methods

All animal experiments were performed under Institutional Animal Care and Use Committee (IACUC) approved protocol (IACUC approval no: 20–129 LF). Eighteen Yorkshire pigs were divided into 3 groups: controls (*n* = 6), MSC group (*n* = 6), and MSC with nanocarrier (MSC + NC, *n* = 6). All animals underwent RFA of liver. The test groups (MSC and MSC + NC) had infusion of MSCs or MSCs with nanocarrier, respectively, via the hepatic artery 24 h after ablation. The control group had no MSC infusion.

### Statistics

Power calculation assumed that the number of ablations within a pig will be four and the expected rates of liver tissue regeneration in MSC and MSC + NC group will be 5% and 85%, respectively. These numbers were chosen according to our previous experience and similar numbers were used in other liver ablation studies [14]. We also assumed that correlation among ablation sites within a pig is 0.5, thus 6 pigs in each group will give 96.3% power to detect difference of 80%. Statistical analysis for all studies was performed using the IBM SPSS software. The independent sample T-test was used for analysis of continuous variables and one-way ANOVA tests were used for categorical variables with significance level set at 0.05.

### Stem Cell Preparation

The procedures for the preparation of nanocarrier-coated stem cells are detailed in Online supplementary materials.

### Animal Interventions

All animal procedures were performed by a board-certified interventional radiologist with swine expertise. RFA was conducted using the Covidien Cool-Tip ablation system (Covidien, Boulder, Colorado) under US guidance on the CT table with general anesthesia. After confirming anesthesia depth, an initial CT scan was done. Based on liver lobe size and anatomy, 2 to 4 ablations of 2 cm diameter were planned per animal. A Covidien Cool-Tip, 11-gage ablation probe was inserted under US guidance, and RFA was performed for 6 min at 80 Watts in impedance control mode. Immediately after, a contrast-enhanced triple-phase CT scan of the liver was obtained using Omnipaque-180 (388 mg of iohexol per mL) for IV contrast (1.4 mL/kg).

The control animals had no additional procedures after ablation. The MSC and MSC + NC groups underwent intraarterial stem cell delivery the day after ablation under general anesthesia. After confirming anesthesia depth, a 5-French vascular sheath was placed in the common femoral artery. A 5-French Cobra catheter was advanced into the abdominal aorta, and a microcatheter (2.8 French Progreat, Terumo, Japan) was used to catheterize the proper hepatic artery. The MSC group received approximately 1 × 10^6^ plain MSCs in 5 mL of PBS, injected into the hepatic artery. The MSC + NC group received approximately 1 × 10^6^ MSCs coated with nanocarriers. Hemostasis was achieved by manual compression after catheter removal.

All animals survived for 30 days, except the first two control animals. One died the day after ablation, and the other was euthanized due to severe distress. Necropsy revealed large areas of ablation but no direct cause of death, likely due to device malfunction related to power settings.

All animals had blood tests (liver function, renal function, and complete blood count) on days 0 (prior to ablation), 15, and 30. On day 30, they received a contrast-enhanced liver CT scan under anesthesia, were euthanized, and underwent necropsy for liver pathology analysis.

The ablation cavity on contrast enhanced CT was defined as the area that does not enhance compared to the rest of the parenchyma. The ablation cavity volume was measured by CT using 3D Slicer (Brigham Women’s Hospital, Boston, MA), a free, open-source medical image computing software [15]. Two board-certified radiologists with at least 5 years of experience independently contoured the ablation cavities. Both reviewers were blinded to the identity of the groups. The reduction in ablation cavity volume at 1 month was used as a surrogate marker of liver regeneration, which was compared between the groups [16].

Cell proliferation at the margins of the ablation cavity was measured by immunohistochemical staining of formalin-fixed paraffin-embedded (FFPE) tissue sections using the proliferation marker Ki67 (Clone K2, Lot# 70,036, Leica Biosystems Inc, Buffalo Grove, IL). The HepPar-1 immunohistochemical stain (Clone OCH1E5, Lot# 69,056, Cell Marque Corp, Rocklin, CA) was used to confirm and localize areas of hepatocyte-specific regeneration. The GFP-transduced cells were identified from FFPE tissue sections using a fluorescent microscope. For all markers, the three areas with the highest count of positive cells (“hotspots”) were selected at medium and high magnifications for each ablation cavity sample. ImageJ software (https://imagej.nih.gov/ij/) quantified positive cells in the three hotspots, and the counts were averaged to obtain a final count per ablation cavity. GFP positivity was graded for all specimens using a 1–4 Likert scale based on GFP positive cells per HPF, as visually analyzed by a blinded pathologist. Likert score was defined as- score 1: < 25% cells/HPF are GFP^+^, 2: 25–50% of cells/HPF are GFP^+^, 3: 50–75% of cells/HPF are GFP^+^, 4: 75–100% of cells/HPF are GFP^+^. A similar Likert scoring was also used for HepPar^+^ cells. All histopathological evaluations were performed by a board-certified pathologist who was blinded to the identity of the groups.

The liver function tests on day 15 and 30 were also compared between test and control groups. Complete blood count, renal function tests, and prothrombin time/international normalized ratio (PT/INR) were monitored for each group to identify any systemic effect or toxicity.

## Results

**Fig. 2:**
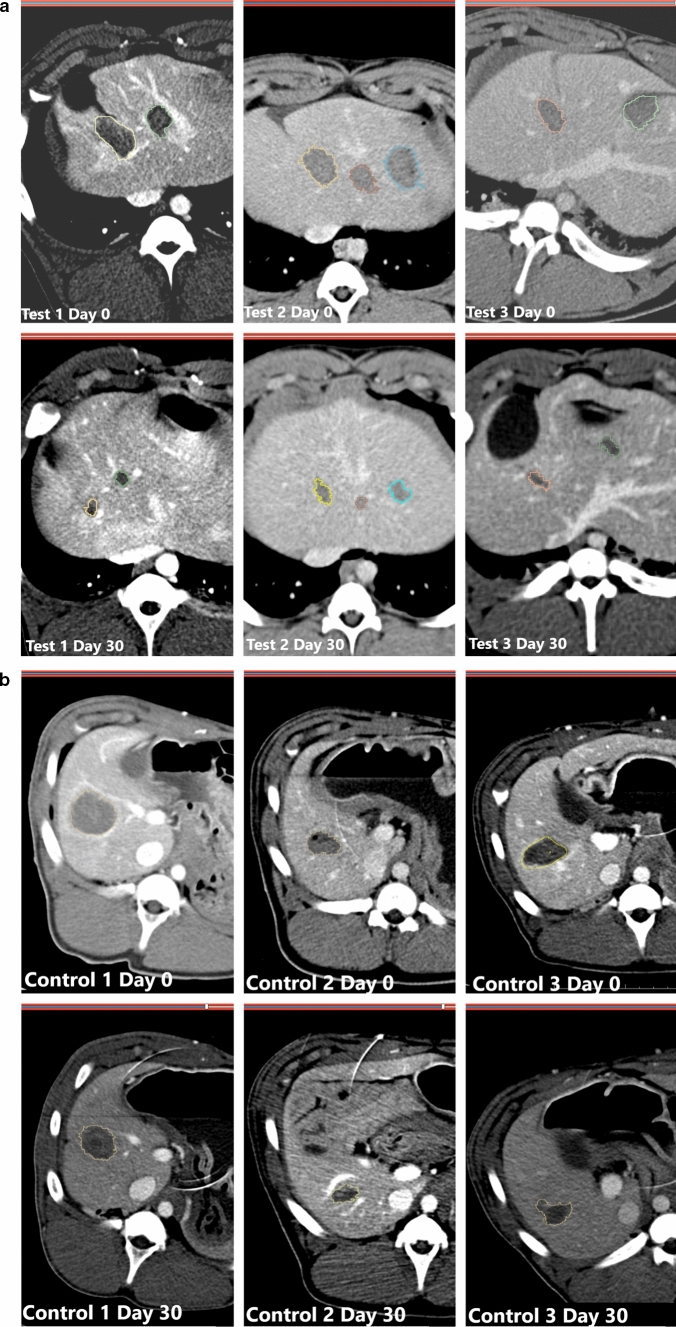
**a** and **b** Comparison of the contrast enhanced CT scan in a test and control animals at 0 and 30 days illustrating the change in the ablation cavity volume. **a** Shows the change in ablation cavity volume from day 0 to day 30 in 3 representative test (MSC + NC) animals. **b** Shows the reduction in ablation cavity volume from day 0 to day 30 in 3 representative control (MSC) animals

**Fig. 3:**
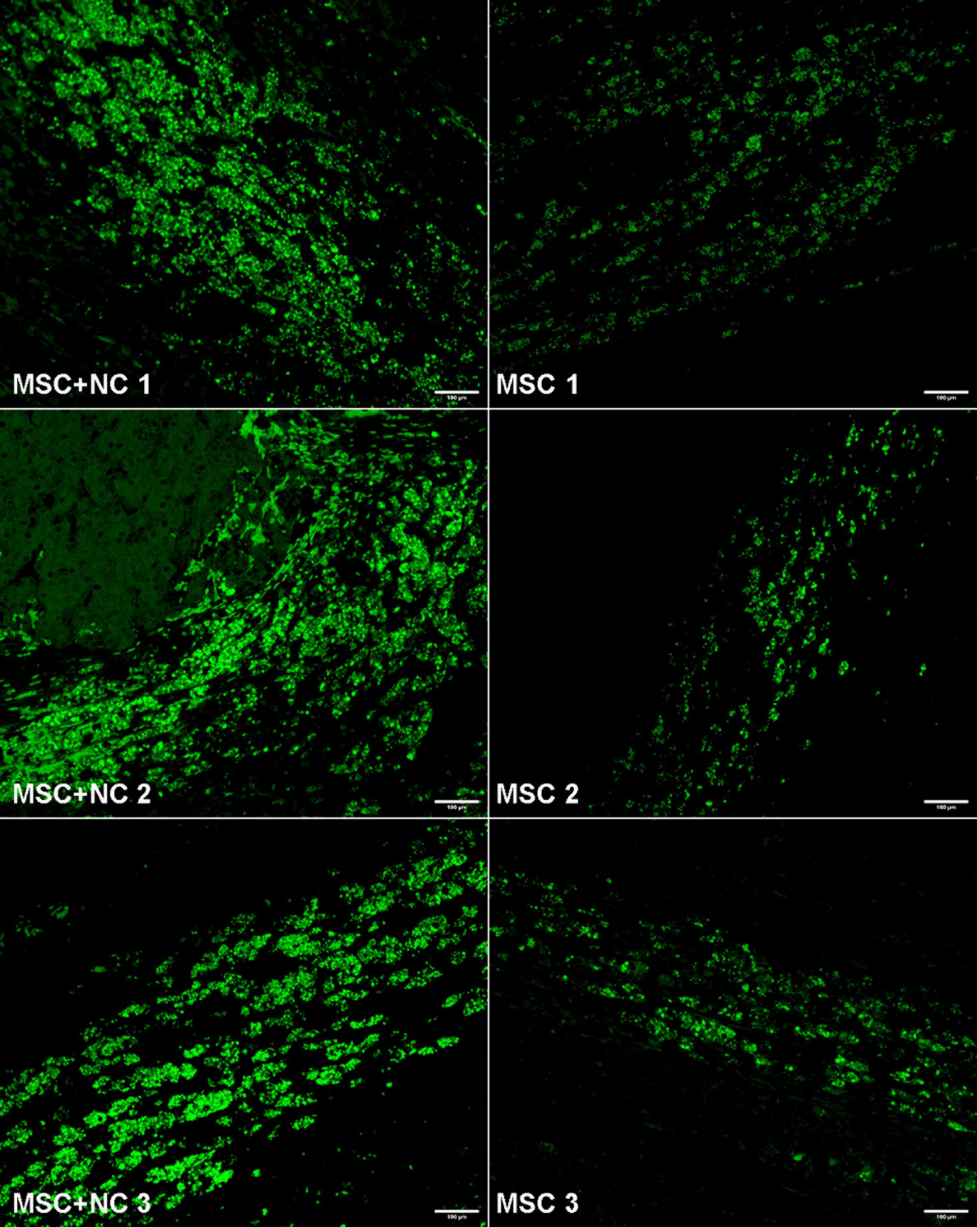
Fluorescent microscopy images from the hot spots at ablation margins from 3 representative test (MSC + NC) and control (MSC) animals. The scale bars correspond to 100 µm

**Fig. 4:**
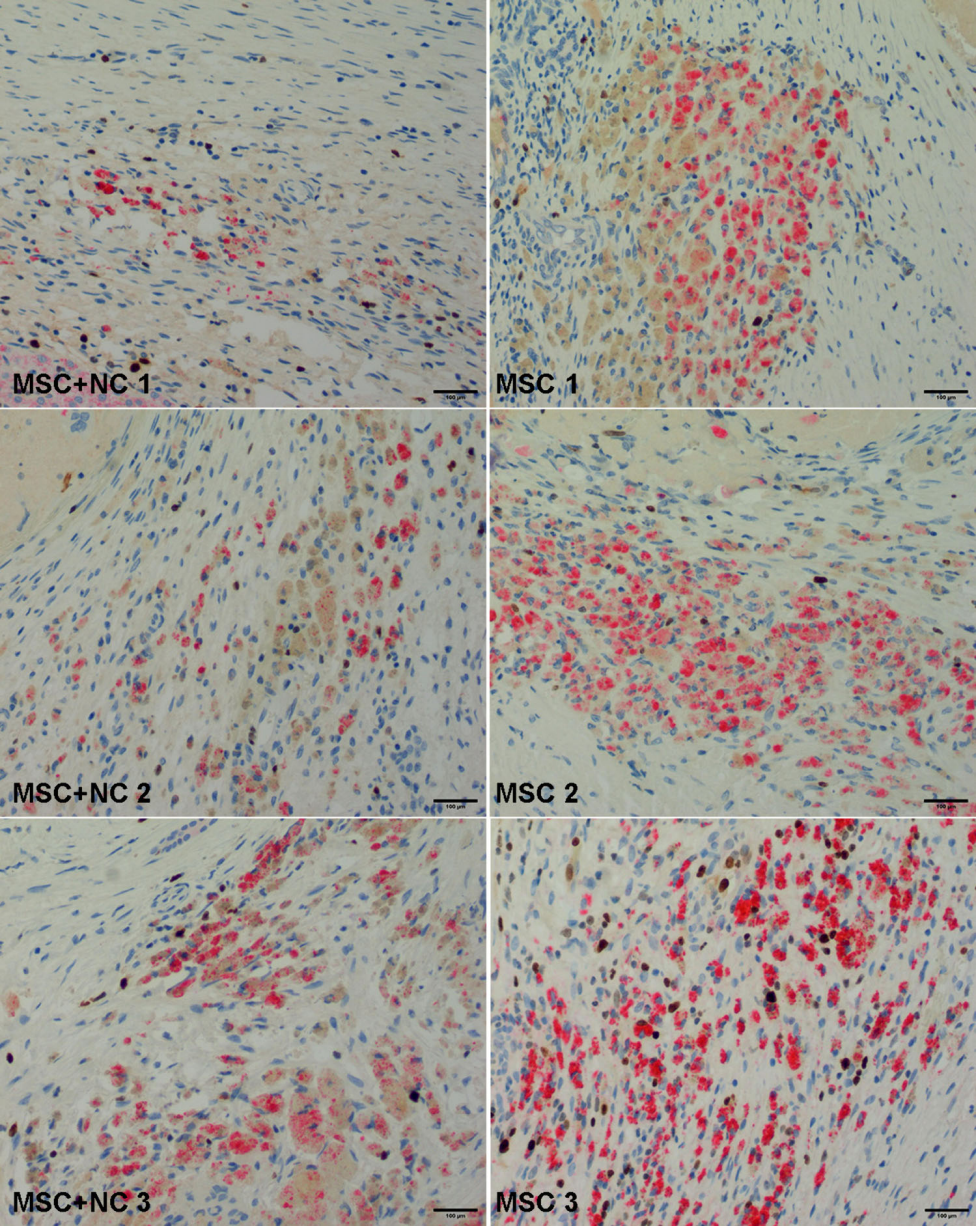
Images of the hotspots from the ablation margins stained with HepPar-1 immunohistochemical stain from the MSC and MSC + NC group. HepPar-1 positive cells are seen with red cytoplasm. The scale bars correspond to 100 µm

**Fig. 5:**
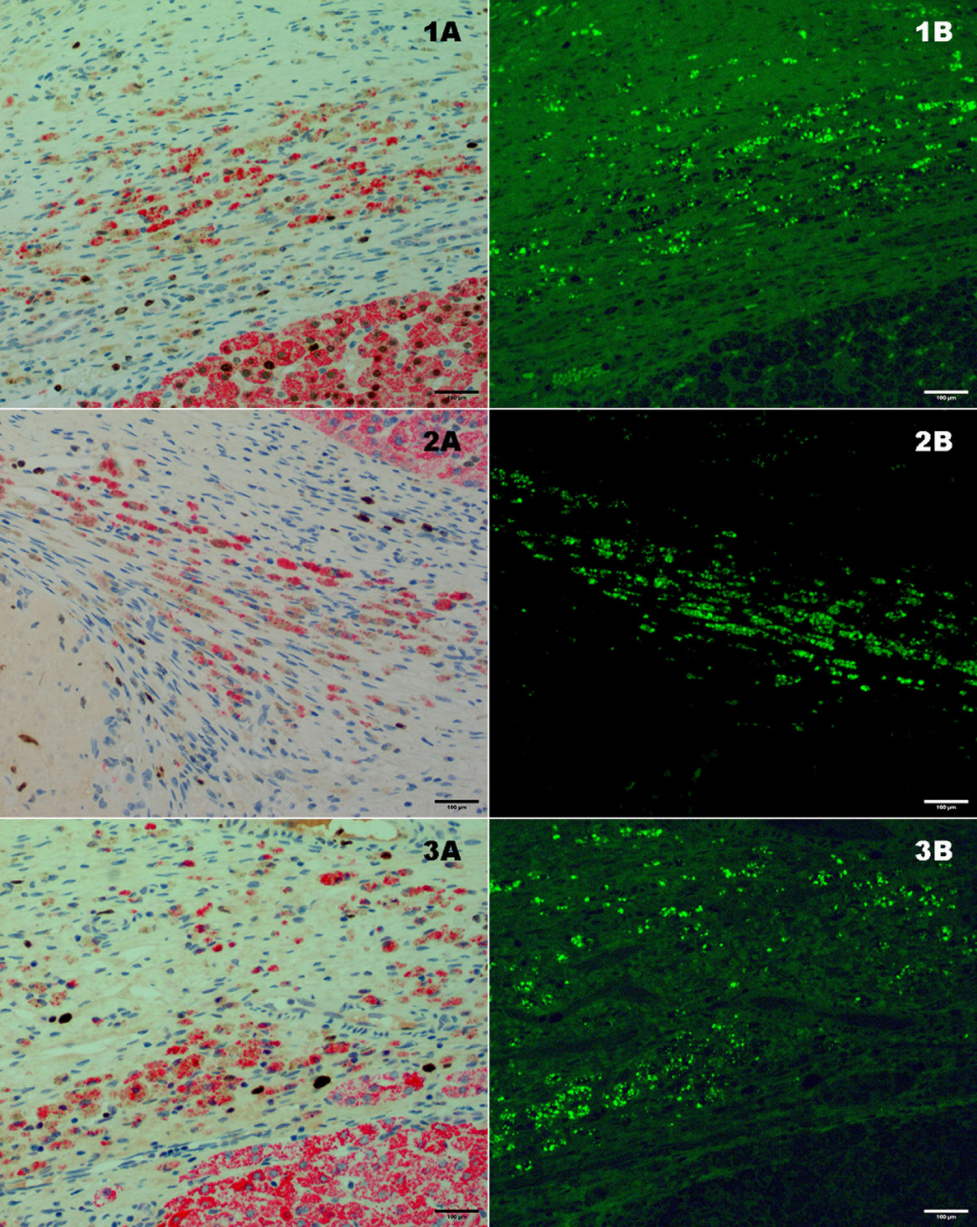
Fluorescent microscopy and HepPar-1 staining from the same hotspots from 3 representative animals showing correlation between HepPar-1 positive (1A, 2A, 3A) and GFP positive (1B, 2B, 3B) cells. The scale bars correspond to 100 µm

The mean weight of control, MSC and MSC + NC group animals were 39.7, 33.5 and 40 kg, respectively. The number of ablations per control, MSC, and MSC + NC groups was 22, 23, and 21, respectively. Two of the control animals died the day after ablation. Therefore, only 13 ablations could be analyzed from the control group. The change in volume of the ablation cavity from day 0 to day 30 on the contrast enhanced CT scan was used as a surrogate to measure the overall tissue regeneration at the ablation margins. There was high degree of correlation between the ablation cavity volume estimated by the two blinded radiologists (Intraclass correlation coefficient = 0.993, *p* < 0.001). The results of the Mesenchymal Stem Cell Therapy are summarized in Table 1 and Fig. 2a–b. Fluorescence emission from the GFP in cells was used for identification of the cells derived from the MSC at the ablation margins (Fig. 3). The Hep Par positive cells per hot spots were also assessed using absolute count as well as a Likert scale of 1 to 4 (Fig. 4). There was also high degree of correlation between the GFP positivity and Hep Par positivity per hotspots (Fig. 5). The overall cellular proliferation at the margins was assessed using Ki67 staining. The mean percentage of Ki67 positive cells count for 3 hot spots per ablation cavity margin was 5.09 ± 3.7% for the MSC group and 6.12 ± 4.2% for the MSC + NC group (*p* = 0.09).

**Table 1:** Results for the targeted MSC therapy

Parameter	Control (*N* = 6)	MSC (*N* = 6)	MSC + NC (*N* = 6)	*p*-valve
Weight	39.7	33.5	40	
# of ablations	22 (13)	23 (23)	21 (21)	
Cavity volume, cm^3^ (day 30)	7830 ± 3903	10,762 ± 8900	7416 ± 4473	0.20
Cavity volume, cm^3^ (day 30)	2542 ± 929	3758 ± 3043	1416 ± 888	
% reduction in cavity volume	64.4 ± 15.0	61.5 ± 12.9	80.3 ± 9.4	< 0.001
eGFP^+^ cell count (Likert scale)	N/A (N/A)	426.8 ± 193.2 (2.58 ± 0.48)	498.6 ± 235.2 (2.80 ± 0.30)	0.01 (0.02)
HEpPar^+^ cell count (Likert scale)	N/A (N/A)	7.59 ± 3.7 (0.57 ± 0.39)	9.81 ± 4.5 (0.97 ± 0.40)	< 0.001 (< 0.001)

There was no significant difference in the liver function, renal function, and complete blood count between the groups at any time point.

## Discussion

Cirrhosis is present in 80–90% of HCC cases, with about half of these patients dying from liver failure complications rather than tumor progression [1, 17, 18]. According to the Barcelona Clinic for Liver Cancer guidelines, patients with Child–Pugh stage C cirrhosis are ineligible for surgical resection or ablation [19]. This study's exploration of liver regeneration using stem cells for liver-directed therapies is thus significant.

A major challenge with stem cell therapy is targeted delivery. Direct injection into the organ is common but may cause issues like tissue pressure, poor oxygenation, and nutrient supply. Systemic delivery via intravenous or intra-arterial routes avoids these issues, but cells can randomly home to various organs [5]. To solve this problem, an innovative method of attaching adhesion molecules (proteins) on MSC surface using nanotechnology has previously been reported [6]. Positively charged nanocarriers complexed with specific adhesion molecules interact with the negatively charged stem cell membrane, coating it through ionic interactions. These nanocarriers enable effective installation of adhesion molecules on the MSC surface, helping the cells engraft to injured tissues by binding with adhesion molecules on the activated endothelial surface.

RF ablation induces a time-dependent immunologic response at the ablation zone perimeter [9], leading to upregulation of ICAM-1 and VCAM-1 in the endothelium [10]. Circulating MSCs complexed with adhesion molecules bind to these endothelial molecules in the inflamed tissue (Fig. 1). Once attached, MSCs undergo trans-endothelial migration, infiltrating the parenchyma [20]. Nanocarriers facilitate MSC docking to the endothelium [6], a critical step in targeted cell delivery. LFA-1, the natural counter-receptor of ICAM-1 [12, 13], has an α-subunit with an I-domain essential for this binding.

Thermal ablation cavity can be clearly visualized as hypodense area in contrast enhanced CT scans given that the dead tissues do not have perfusion [21, 22]. The ablation cavity can be followed up with contrast CT and the change in the volume of the ablation cavity can be accurately measured, which is an indirect marker of liver tissue regeneration at the margins [16]. This study shows a significantly better ablation cavity volume reduction for animals which received MSCs with nanocarriers compared to those which received plain MSCs or no MSCs. These findings confirm better liver regeneration at the ablation margins when targeting nanocarrier was used in conjunction with the MSCs.

**Fig. 6:**
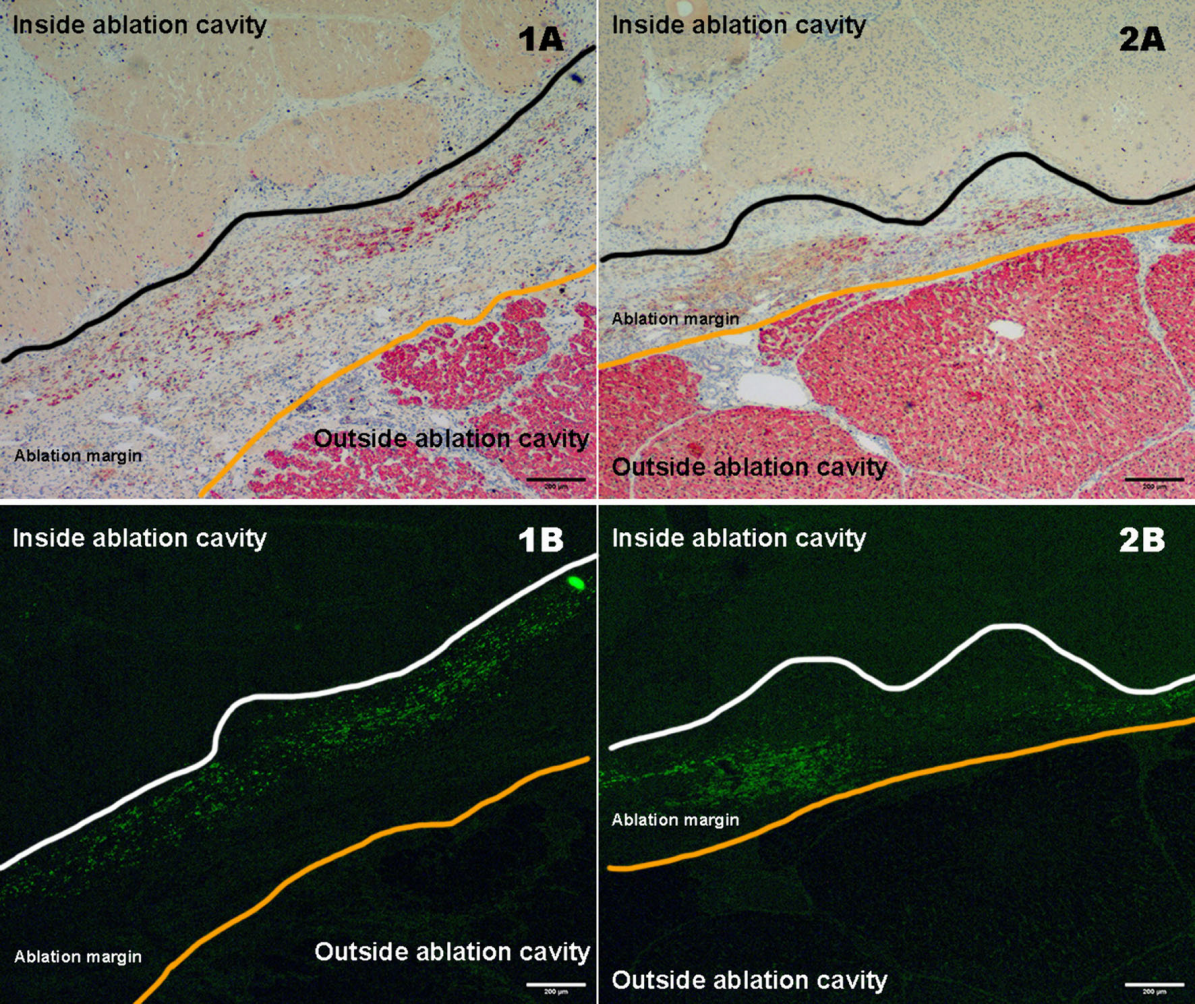
Low power light and fluorescent microscopy images from the ablation margins in the MSC + NC group showing GFP + ve MSCs deposited along the ablation margins. The scale bars correspond to 200 µm

At a microscopic level, MSCs are distributed at the ablation margins in the nanocarrier group, as indicated by eGFP presence showing successful targeted delivery of MSCs to the inflamed peri-ablation margins (Fig. 6). Additionally, there is a higher number of hepatocyte regeneration at these margins in the nanocarrier group. The high correlation between GFP positivity and Hep Par positivity confirms that the hepatocytes originated from MSCs.

Comparing overall cellular proliferation at the margins using Ki67 showed no significant difference between the groups. This may be due to Ki67 being a nonspecific indicator that includes proliferation of inflammatory cells, expected at ablation margins in all groups.

For patients unable to undergo liver resection or major ablations due to potential liver failure, stem cells offer an attractive strategy for regenerating liver tissue. This approach can make these patients eligible for curative treatments like ablation or surgery. MSCs are ideal for liver regeneration, having been shown to differentiate into hepatocyte-like cells with functional properties mimicking hepatocytes [23–25]. Early human trials have explored MSCs in liver disease, demonstrating improved liver regeneration following resection and chemoembolization [26]. A notable study using autologous CD133 + bone marrow stem cells in patients undergoing portal vein embolization before liver resection showed a significantly higher increase in liver volume, enabling earlier surgery for hepatic metastases resection [27].

Nanocarrier-targeted delivery of stem cells is a versatile platform for regenerative medicine. Inflammation at surgical margins after partial organ resection is similar to ablation margins, allowing for targeted stem cell delivery. This strategy can regenerate solid organs like the kidney and pancreas post-ablation or resection. Nanocarriers can also deliver various cells and agents to specific tissues based on selected protein pairs.

A major limitation of the study is that interventions were conducted on normal porcine livers, not cirrhotic ones. Future studies should use cirrhotic animal models. Biodistribution studies were not conducted; however, due to MSCs being delivered locally via the hepatic artery to the ablation site, it is expected that most MSCs will localize there. Further studies in large animal cirrhosis models are necessary before advancing to human studies.

## Conclusion

In conclusion, nanocarrier-mediated MSC therapy enables targeted delivery to ablation margins, promoting liver regeneration shown by reduced ablation cavity volume and increased hepatocyte regeneration in a swine model. This technology could extend liver ablation eligibility to patients with severe liver dysfunction and may also be adapted for regenerating other solid organs post-ablation or resection.

## Supplementary Information

Below is the link to the electronic supplementary material.

Supplementary file1 (DOCX 20 KB)

## Abbreviations

Ac-G5: Acetylated dendrimers
FFPE: Formalin-fixed paraffin-embedded
GFP: Green fluorescent protein
HCC: Hepatocellular cancer
ICAM-1: Intercellular adhesion molecule-1
LFA-1: Lymphocyte function-associated antigen-1
MSC: Mesenchymal stem cells
NC: Nanocarrier
PBS: Phosphate-buffered saline
PMAM: Poly(amidoamine)
RFA: Radiofrequency ablation
