# Supplementary material for: Liver Regeneration Following Thermal Ablation Using Nanocarrier Mediated Targeted Mesenchymal Stem Cell Therapy
Source: Cardiovasc Intervent Radiol. 2024 Nov 6;48(2):233–43. doi: 10.1007/s00270-024-03862-2 (PMC11790787; doi:10.1007/s00270-024-03862-2)
Supplement: Supplementary file 1 — Supplementary file1 (DOCX 20 KB) [file 270_2024_3862_MOESM1_ESM.docx]

**SUPPLEMENTARY MATERIAL**

**Stem Cell Preparation:**

Bone marrow aspirate was obtained from the long hind bone of two donor animals (Yorkshire swine, male and female, age 6 months) using a standard aspiration kit. The cells from the 2 pigs were combined at 1:1 ratio. The aspirates collected in heparin-coated glass tubes were washed with phosphate buffered saline (PBS) twice. Total of 107 Bone marrow (BM) cells were cultured in 10 cm petri-dish with Dulbecco's Modified Eagle Medium/Nutrient Mixture F-12 containing 20% Fetal Bovine Serum and Penicillin/Streptomycin. Non-adherent cells were moved by periodical changes of medium (every 3 days). In about 10 days when cells grew and reached about 70% confluency, MSC were transduced with eGFP/Lentivirus. The pseudotyped lentivirus was produced by co-transfecting 293 T cells with three plasmids as described (Balint K, et al., Activation of Notch1 signaling is required for beta-catenin-mediated human primary melanoma progression. J Clin Invest 2005, 115:3166-3176). To infect MSCs by lentiviruses, cells were exposed for six hours to virus with MOI (multiplicity of infection) 5 in the presence of 4 mg/mL polybrene (Sigma-Aldrich). Cells were then rinsed with regular complete medium and cultured with regular complete medium for two additional days. The eGFP+ MSCs were sorted by fluorescence activated cell sorter (FACS). Two days after transduction, eGFP+ MSCs were detached from culture dishes by Trypsin-EDTA and washed twice by PBS and were re-suspended in complete DMEM/F-12. The concentration of DMEM was 100 U/mL (1:100 dilution from stock solution which is 10,000 U/mL).

**Nanocarrier Preparation and Conjugation:**

**G5-dendrimers nanoparticles**

G5-dendrimers (5th generation polyamidoamine (PAMAM) dendrimers, Dendritic, Midland, MI) are 5th generation polyamidoamine dendrimers that has an ethylenediamine core and a repeating amidoamine structure, which has terminal primary amines that are positively charged at physiological pH and can be modified. They are grown off the ethylenediamine core in an iterative synthesis, each step requiring a new generation of the dendrimer. Therefore, fifth generation PAMAM dendrimers have 128 theoretical amine groups that can be modified with a measured diameter of ~50 Å and theoretical molecular weight of 28,826 Da.

**Acetylated Dendrimers (Ac-G5)**

The acetylated dendrimers (Ac-G5) were used for the preparation of the nanocarriers. The ratio between the acetic anhydride and the dendrimer was adjusted so that about 30% of the total amine groups would be acetylated to reduce the surface charge density and reduce cytotoxicity. The amount of acetic anhydride was calculated on the basis of the number of primary amines as reported by the manufacturer. Triethylamine (10% excess based on the amount of acetic anhydride) was added to quench the acetic acid formed as a side product during the reaction. The reactions were carried out in a glass flask in anhydrous methanol solution at room temperature for 24 h. The reaction mixture was dialyzed first in phosphate buffer at pH 8.0 and then in deionized water.

Briefly, to 15 mL of anhydrous methanol in a magnetically stirred round bottom flask, 1 mL of 5 wt% PAMAM (0.05 g, 1.73 µmol, 1.0 eq) in methanol was added. Triethylamine (6.8 µL, 48.71 µmol, 28.15 eq) was added to the flask and stirred for 30 minutes. Then, acetic anhydride (4.2 µL, 44.29 µmol, 25.6 eq) was added dropwise to the reaction mixture and the reaction was carried out overnight at room temperature under an argon atmosphere in a Schlenk line. The methanol was then removed by rotary evaporator, and the residue was dissolved in distilled water. The dendrimer solution was then dialyzed against 1 L of phosphate buffered saline (PBS) for 8 hours followed by extensive dialysis against water (3 x 8 h) in a 10,000 kDa cutoff Slide-A-Lyzer Dialysis cassette (Pierce Biotechnology, Rockford, IL). The samples were then lyophilized and stored at 4 °C.

Expression of the I-domain of LFA-1

The expression of the I-domain of LFA-1 has been performed as previously has been described [19] with minor modifications. A plasmid containing the I-Domain DNA sequence, pET-11a/LFA-1, was transformed into competent E. coli BL21 cells. The cells were grown in 5.0 mL LB Broth containing 100 µg/mL ampicillin. Next day, 300 mL of LB Broth containing 100 µg/mL ampicillin was inoculated with 5.0 mL of the refreshed overnight cultures. To refresh, the overnight cultures were centrifuged at 5,000 xg for 10 minutes. The spent LB broth was discarded and the cell pellet was resuspended in the same volume of fresh LB broth. The flasks were incubated at 37 °C, with shaking at 250 rpm, until the optical density of the culture at 600 nm (OD600) was around 0.8. Then, 300 µL of 1.0 M IPTG and 3.0 mL of 1.0 M magnesium chloride was added to have final concentrations of 1.0 mM and 10.0 mM, respectively. The flask was incubated at room temperature for 3.0 h, with shaking at 250 rpm. The culture was then centrifuged at 8,000 xg for 15 minutes at 4 °C to harvest the bacterial cells expressing LFA-1 in the form of inclusion bodies. The LB broth was discarded, and the cell pellet was resuspended in 30 mL of homogenization buffer (50 mM TRIS buffer containing 1.0 mM DTT, 10 mM magnesium chloride, 2.0 mM EDTA, and 5.0 mM benzamidine hydrochloride at pH 8.00) and sonicated using 1 s pulses for 20 minutes at a 20% amplitude. After the sonication is complete, the cell suspension was centrifuged at 18,000 xg at 4 °C for 15 minutes. Then the pellet was washed by resuspending in 30 mL of homogenization buffer and centrifuging at 18,000 xg at 4 °C for 15 minutes. This washing was performed once more, followed by a final wash using deionized water. The pellet, which is consisting of LFA-1 I-domain in the form of inclusion bodies, was solubilized in about 30 mL of denaturation buffer (50 mM TRIS buffer containing 2.0 mM DTT, and 6 M guanidine hydrochloride at pH 8.00) by rotating at room temperature for 1.0 h. Then, the solution was centrifuged at 18,000 xg at 4 °C for 15 minutes to remove any insoluble matter. The solution was diluted in 200 mL cold DI water at 4 °C followed by rapidly diluting in 4.0 L of cold renaturation buffer (50 mM TRIS buffer containing 1.0 mM DTT, and 5 mM magnesium sulfate and 7% glycerol at pH 8.00) to refold the LFA-1 I-Domain protein into its functional form. This solution contains dilute, purified, LFA-1 I domain which was then concentrated using 3,500 MWCO tangential flow filtration (TFF) column. After concentrating the protein sample 40-fold, the sample was dialyzed using 10,000 MWCO dialysis cassettes into phosphate buffered saline at pH 7.4. Any formed precipitate during dialysis was removed by centrifuging at 18,000 xg at 4 °C for 15 minutes. The purity of the protein was confirmed using sodium dodecyl sulfate polyacrylamide gel electrophoresis (SDS-PAGE), followed by the measurement of the protein concentration using BCA assay. The isoelectric point of the purified protein was determined using Novex IEF Gel pH, 3-10 according to the manufacturer’s protocol.

**Determination of optimal cell surface coating conditions**

The optimal conditions for the preparation of the G5-dendrimer as nanocarriers for targeted delivery has been previously reported (Jun-Zhao Liu, et al., Directing and Potentiating Stem Cell-Mediated Angiogenesis and Tissue Repair by Cell Surface E-Selectin Coating” PLoS One, 2016, 11(4), 1-18). Acetylating ~30% of the positively charged amines was determined as the optimal condition that decreases the overall positive charge of the G5-dendrimer while retains cell surface binding capability, to prolong cell surface coating duration and avoid internalization of the nanocarrier into the coated cells. Optimization of the complexation protocol prolonged cell surface coating up to 3-hours, which was confirmed by transmission electron microscopy (TEM) and confocal fluorescence experiments. Z-stacks showed that Ac-G5-albumin-FITC nanocarriers stayed on the surface of MSC for 3-hours with minimal internalization. To minimize cellular toxicity, optimal dendrimer modification conditions and ratio of dendrimer to protein to be conjugated were determined. Then, the cytotoxicity of various dendrimers and modification conditions toward human umbilical vein endothelial cells (HUVEC) was examined. Cell viability assays demonstrated that Ac-G5 nanocarriers with 30% acetylation did not cause HUVEC cytotoxicity.

**Complexing LFA-1 fragments to the Ac-G5 dendrimers**

In order to prepare the LFA-1 I-Domain/PAMAM G5 Dendrimer complex, 1750 µL of 1.0 mg/mL solution of LFA-1 I-domain in PBS was mixed with 1250 μL of OPTIMEM medium. Meanwhile, an aliquot of 474 μL of 10 mg/mL solution of 30% acetylated, Ac-G5 dendrimer was dissolved in 2526 μL OPTIMEM medium. The prepared dendrimer solution was added to the LFA-1 I-Domain solution while mixing (Total Volume= 6000 μL). The resulting solution was incubated at room temperature for 15 minutes. Any formed precipitate during incubation was removed by centrifuging at 18,000 xg at 4 °C for 15 minutes. The nanocarriers were then washed and re-suspended with DMEM.

**MSC and nanocarrier conjugation**

To coat the cells, 1 mL of nanocarriers were mixed with 1x 106 MSC and was incubated for 20 min at room temperature with gentle mixing every 5 minutes. Afterwards, nanocarriers-coated MSC were centrifuged at a force of 270 x g for 5 minutes and were gently re-suspended with pipette in 5 mL sterile PBS. MSC(CD73+/CD44+/CD105+) were characterized by flow cytometry.
